# The Synthetic Lignan Secoisolariciresinol Diglucoside Prevents Asbestos-Induced NLRP3 Inflammasome Activation in Murine Macrophages

**DOI:** 10.1155/2017/7395238

**Published:** 2017-09-13

**Authors:** Ralph A. Pietrofesa, Patrick Woodruff, Wei-Ting Hwang, Priyal Patel, Shampa Chatterjee, Steven M. Albelda, Melpo Christofidou-Solomidou

**Affiliations:** ^1^Division of Pulmonary, Allergy, and Critical Care Medicine and the Department of Medicine, University of Pennsylvania Perelman School of Medicine, Philadelphia, PA 19104, USA; ^2^Department of Biostatistics and Epidemiology, University of Pennsylvania Perelman School of Medicine, Philadelphia, PA 19104, USA; ^3^Institute for Environmental Medicine, Department of Physiology, University of Pennsylvania Perelman School of Medicine, Philadelphia, PA 19104, USA

## Abstract

**Background:**

The interaction of asbestos with macrophages drives two key processes that are linked to malignancy: (1) the generation of reactive oxygen species (ROS)/reactive nitrogen species (RNS) and (2) the activation of an inflammation cascade that drives acute and chronic inflammation, with the NLRP3 inflammasome playing a key role. Synthetic secoisolariciresinol diglucoside (SDG), LGM2605, is a nontoxic lignan with anti-inflammatory and antioxidant properties and was evaluated for protection from asbestos in murine peritoneal macrophages (MF).

**Methods:**

MFs were exposed to crocidolite asbestos ± LGM2605 given 4 hours prior to exposure and evaluated at various times for NLRP3 expression, secretion of inflammasome-activated cytokines (IL-1*β* and IL-18), proinflammatory cytokines (IL-6, TNF*α*, and HMGB1), NF-*κ*B activation, and levels of total nitrates/nitrites.

**Results:**

Asbestos induces a significant (*p* < 0.0001) increase in the NLRP3 subunit, release of proinflammatory cytokines, NLRP3-activated cytokines, NF-*κ*B, and levels of nitrates/nitrites. LGM2605 significantly reduced NLRP3 ranging from 40 to 81%, IL-1*β* by 89–96%, and TNF*α* by 67–78%, as well as activated NF-*κ*B by 48-49% while decreasing levels of nitrates/nitrites by 85–93%.

**Conclusions:**

LGM2605 reduced asbestos-induced NLRP3 expression, proinflammatory cytokine release, NF-*κ*B activation, and nitrosative stress in MFs supporting its possible use in preventing the asbestos-induced inflammatory cascade leading to malignancy.

## 1. Introduction

Recent studies have indicated that the pathogenesis of asbestos-induced cancers involves chronic inflammation which is facilitated by the cytokines interleukin-1 beta (IL-1*β*), the chemokine tumor necrosis factor alpha (TNF*α*), and high mobility group box-1 (HMGB1) and eventual oxidative tissue damage caused by persistent asbestos fibers [1, 2]. Inhaled asbestos fibers permeate into the lung and ultimately to the pleural surface, where they are taken up by tissue phagocytes, primarily macrophages [3, 4]. Macrophages exposed to asbestos then undergo frustrated phagocytosis of elongated fibers [5]. Frustrated phagocytosis of asbestos fibers by macrophages and mesothelial cells generates intracellular reactive oxygen species (ROS) and reactive nitrogen species (RNS) which, besides being deleterious due to direct oxidative damage, also activate proinflammatory transcription factors such as NF-*κ*B, leading to the generation of numerous proinflammatory cytokines. Furthermore, oxidants and inflammatory moieties contribute to DNA damage and ultimately lead to malignant transformation of mesothelial cells [6]. Asbestos-activated macrophages also contribute to tumorigenesis by overproduction of ROS/RNS that can in turn induce further DNA damage and lead to potential genomic instability [7]. Work by Yang and coworkers expanded the old hypothesis of ROS-induced tumorigenesis to attribute a key role for HMGB-1 in this process [8].

Inflammation plays a key role in the pathology of asbestos-induced lung cancers; indeed, asbestos-induced inflammation is considered to be a critical event in the development of malignant mesothelioma (MM) [9, 10]. This inflammation has largely been attributed to the activation of the NF-*κ*B and subsequent induction of inflammatory genes. Lately, there is increasing evidence of a role for the activated NLRP3 inflammasome in sustaining and amplifying inflammation caused by asbestos [11–13].

The NLRP3 inflammasome is part of the innate immune system. The NLRP3 subunit is a receptor on immune and other cells and forms a macromolecular complex in response to external stimuli. This complex, which is comprised of the NLRP3 subunit and adaptor proteins, functions as a scaffold for inactive caspase-1 that is activated upon binding to the inflammasome. Active caspase-1 cleaves the proinflammatory IL-1 family of cytokines into their bioactive forms, IL-1*β* and IL-18. These active cytokines are primary drivers of cell death and inflammation. We have shown in our previous work [14] that LGM2605 inhibits asbestos-induced cell death in murine macrophages.

Thus, a well-tolerated and safe agent with anti-inflammatory properties that targets the NLRP3 inflammasome could potentially be used to prevent the onset of inflammation signals that lead to the development of malignant mesothelioma (MM) in asbestos-exposed populations. MM is on the rise across the US and Western Europe [15] with more than 7000 reported cases annually. This necessitates the identification of a protective agent which can block the pathology of MM.

Previous studies in various models of inflammation-induced lung disease, including ischemia/reperfusion [16] and radiation-induced fibrosis [17], suggested that the flaxseed lignan secoisolariciresinol diglucoside (SDG) has these requisite anti-inflammatory and antioxidant properties [18, 19]. We thus hypothesized that SDG or an SDG-rich flaxseed lignan component (FLC) formulation administered via the diet might be useful in the chemoprevention of asbestos-induced malignant mesothelioma and have begun a series of studies to test the validity of this idea. Earlier, we carried out an *in vivo* study in which we evaluated the usefulness of an FLC-supplemented diet in a murine model of acute asbestos-induced peritoneal inflammation. Three days after intraperitoneal instillation of asbestos into mice, we observed both inflammation and oxidative/nitrosative stress in the peritoneal fluid. The FLC diet led to marked reductions in total white blood cell influx and proinflammatory IL-1*β*, IL-6, TNF*α*, and HMGB1 cytokine release [20].

These findings indicated a protective role for SDG-rich formulations in asbestos-induced inflammation; however, the mechanisms or cell types that conferred this protection were not clear. The contribution of the main flaxseed lignan, that is, the purified compound SDG, was also not known. The effect of SDG in combating asbestos-induced inflammation signaling needs to be studied to facilitate the use of SDG as a preventive or protective agent against asbestos-induced lung damage. SDG was chemically synthesized (LGM2605) by a proprietary pathway [18], to enable evaluation through animal testing in anticipation of eventual clinical usefulness. Synthetic secoisolariciresinol diglucoside (SDG), LGM2605, is a nontoxic lignan with anti-inflammatory and antioxidant properties and was evaluated for protection from asbestos in murine peritoneal macrophages (MF). LGM2605 was found to be similar to natural SDG (extracted from whole grain flaxseed), acting as a free radical scavenger and an antioxidant, with DNA-protective activity [19]. Importantly, our recent study also found that LGM2605 possessed potent cell protective properties [21] and, when tested on asbestos-activated elicited murine macrophages, it induced cell protective defenses, such as cellular Nrf2 activation and the expression of phase II antioxidant enzymes, HO-1 and Nqo1, and reduced asbestos-induced ROS generation and markers of oxidative stress [14]. Thus, we carried out the present study to determine whether inhibition of inflammasome activation was implicated in the mechanism of LGM2605 protection from asbestos exposure of macrophages. Our objectives were (1) to characterize the inflammatory pathway triggered in murine peritoneal macrophages following asbestos exposure, (2) to evaluate the effect of LGM2605 in modulating this inflammation, and (3) to evaluate the chemopreventive properties of LGM2605 by determining whether it acts via inhibition of asbestos-induced inflammasome activation.

## 2. Materials and Methods

### 2.1. Harvesting of Murine Peritoneal Macrophages

Murine peritoneal macrophages (MF) were harvested from the peritoneum following elicitation using thioglycollate broth according to the method described by Zhang et al., [22] whereby a uniform MF population is obtained. Mice were used at 13 weeks of age under animal protocols approved by the Institutional Animal Care and Use Committee (IACUC) of the University of Pennsylvania (Philadelphia, PA). Animals were housed in conventional cages under standardized conditions with controlled temperature and humidity, and a 12-12-hour day-night light cycle. Animals had free access to water and mouse chow. Mice were injected, via intraperitoneal (IP) injection, with 1 ml of a 3% solution of thioglycollate broth in 0.5 ml phosphate-buffered saline (PBS). Three days following thioglycollate exposure, mice were euthanized using an overdose of ketamine (160 mg/kg) and xylazine (25 mg/kg). Peritoneal lavage (PL) was then performed through a 20-gauge angiocatheter (BD Pharmingen, San Diego, CA, USA), with the intraperitoneal instillation of 3 ml Hank's balanced salt solution (HBSS, Ca^2+^, Mg^2+^ free). An aliquot of peritoneal lavage fluid (PLF) was immediately separated to measure total cell counts (cells/ml PLF) using a Coulter Cell and Particle Counter (Beckman Coulter, Miami, FL, USA). Murine peritoneal macrophages were pooled and plated in 1 ml of cell culture medium (phenol-free RPMI supplemented with 1% FBS and supplemented with penicillin (100 units/ml) and streptomycin (100 *μ*g/ml) and L-glutamine (2 mm)) in a 6-well plate (2 × 10^6^ cells/well) and allowed to adhere to the bottom of the wells. Overall, 10–20 million cells are obtained from each mouse. Elicited peritoneal macrophages were used to determine the effects of LGM2605 in preventing asbestos-induced inflammasome activation, cytokine secretion, antioxidant response, and asbestos-induced cytotoxicity.

### 2.2. Crocidolite Asbestos Exposure

Elicited peritoneal macrophages were exposed to sterile UICC crocidolite (SPI Supplies, West Chester, PA, USA) asbestos fibers that were baked overnight, resuspended in 1X PBS at a stock concentration of 800 *μ*g/ml, and sonicated for 30 minutes. The solution of asbestos fibers was exposed to ultraviolet light prior to use in cell culture experiments. For all experiments, murine peritoneal macrophages were exposed to crocidolite asbestos fibers at a concentration of 20 *μ*g/cm^2^ based on our previous studies [14].

### 2.3. LGM2605 Exposure

**Figure 1:**
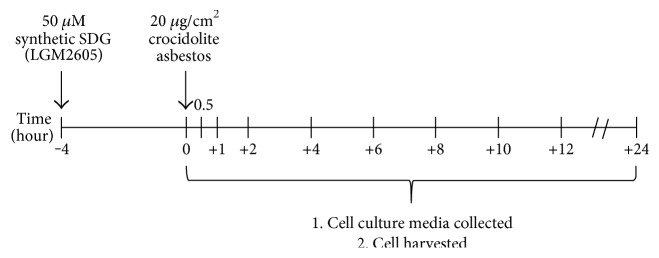
The LGM2605 pretreatment regimen used in this study. Macrophages subjected to asbestos exposure with or without LGM2605. Elicited murine peritoneal macrophages were exposed to 50 *μ*M LGM2605 4 hours prior to exposure to crocidolite asbestos fibers (20 *μ*g/cm^2^). Culture medium and cells were harvested at 0, 0.5, 1, 2, 4, 6, 8, 10, 12, and 24 hours post asbestos exposure.

Chemical synthesis of secoisolariciresinol diglucoside has been previously described [18]. Briefly, LGM2605 was synthesized from vanillin via secoisolariciresinol and a glucosyl donor (perbenzoyl-protected trichloroacetimidate under the influence of TMSOTf) through a concise route that involved chromatographic separation of diastereomeric diglucoside derivatives. LGM2605 was reconstituted to a stock concentration of 10 mM, and cells were exposed to 50 *μ*M LGM2605 4 hours prior to asbestos exposure (see Figure 1()). The 50 *μ*M dose of LGM2605 exposure was selected based on an earlier study in which this dose was sufficient to diminish asbestos-induced ROS generation by macrophages to levels that were comparable to naïve macrophages [14].

### 2.4. Microscopic Visualization of the NLRP3 Subunit of the Inflammasome in Murine Macrophages by Fluorescence Imaging

Elicited murine peritoneal macrophages exposed to crocidolite asbestos fibers at a concentration of 20 *μ*g/cm^2^ were assessed for induction of the NLRP3 inflammasome 24 hours following asbestos exposure. This was done by fixing the cells (1 : 1 methanol-acetone fixation) and immunostaining for the NLRP3 subunit by using polyclonal anti-NLRP3 primary antibody (catalogue number 15101S, Cell Signaling Technology, Danvers, MA, USA) and the goat anti-rabbit-Alexa 488 secondary antibody (Abcam, Cambridge, MA, USA) followed by imaging on a Zeiss LSM510 scanning laser microscope. All images were acquired at the same exposure and offset settings using LSM Metamorph Imaging® software (Molecular Devices, Sunnyvale, CA, USA). The fluorescent images of cells were processed and quantitated for NLRP3 expression by the use of ImageJ software (NIH). The intensity of cells in each field was integrated to obtain the total fluorescence intensity of a particular field. Three to four fields were imaged for each condition (control, LGM2605 only, asbestos treated, asbestos, and LGM2605) for *n* = 3 independent experiments.

### 2.5. Determination of Asbestos-Induced Proinflammatory Cytokine Release from Murine Peritoneal Macrophages

Levels of proinflammatory cytokines, IL-1*β*, IL-6, IL-18, tumor necrosis factor alpha (TNF*α*), and high mobility group box 1 (HMGB1), were determined in cell culture medium at multiple time points post asbestos exposure (0, 0.5, 1, 2, 4, 6, 8, 12, and 24 hours post asbestos) using enzyme-linked immunosorbent assays (ELISA). Samples were run undiluted in triplicate, and assays were performed according to manufacturer's instructions. Levels of IL-1*β*, IL-6, IL-18, and TNF*α* are reported as picograms per milliliter (pg/ml) of culture medium, and levels of HMGB1 released into the culture medium are reported as nanograms per milliliter (ng/ml). ELISA kits (TNF*α* and IL-1*β*) were purchased from BD biosciences (San Jose, CA, USA), MBL International (Woburn, MA, USA) (mouse IL-18 ELISA Kit), R&D systems (Minneapolis, MN, USA) (mouse IL-6 Quantikine ELISA Kit), and Chondrex Inc. (Redmond, WA, USA) (HMGB1 Detection Kit).

### 2.6. Analysis of Nitrate/Nitrite Levels in Cell Culture Medium

Levels of nitrates and nitrites, metabolites of nitric oxide, in the culture medium were determined using a nitrate/nitrite colorimetric assay kit (Cayman Chemical, Ann Arbor, MI, USA) according to the manufacturer's protocol. The assay kit quantifies levels of total nitrates/nitrites (stable breakdown products of nitric oxide) by first converting nitrates to nitrites using nitrate reductase and then measuring total nitrites by adding Greiss Reagent to the reaction mixture, which produces a purple azo compound in the presence of nitrites that can be measured spectrophotometrically. The absorbance of the azo chromophore was measured at 540 nm measured using a SpectraMax i3x Multi-Mode microplate reader (Molecular Devices, Sunnyvale, CA, USA). Cell culture medium samples were run undiluted, and the data are reported as the concentration (*μ*M) of total nitrate/nitrites in the cell culture medium.

### 2.7. NF-*κ*B Transcription Factor Analysis

The presence of nuclear factor kappa-light-chain-enhancer of activated B cells (NF-*κ*B) p65 subunit was determined in nuclear extracts isolated from macrophages exposed to asbestos and harvested at 1, 2, 4, 6, 8, and 12 hours post asbestos exposure. Cytoplasmic and nuclear extracts were prepared using a commercially available nuclear extraction kit (Cayman Chemical, Ann Arbor, MI, USA). Transcription factor assay kits (Cayman Chemical, Ann Arbor, MI, USA) were used to detect nuclear NF-*κ*B. The transcription factor assay kits utilize a specific double-stranded DNA sequence containing the NF-*κ*B response element. The data are reported as the ratio of the absorbance at 450 nm (OD_450_) to the protein extract concentration (*μ*g).

### 2.8. RNA Isolation and Gene Expression Analysis

Total RNA was isolated from murine peritoneal macrophages using a commercially available kit, RNeasy Plus Mini Kit, supplied by Qiagen (Valencia, CA, USA). Total RNA was quantified using a NanoDrop 2000 apparatus (ThermoFisher Scientific, Waltham, MA, USA). Reverse transcription of RNA to cDNA was then performed on a Veriti® Thermal Cycler using the high capacity RNA to cDNA kit supplied by Applied Biosystems. Quantitative polymerase chain reaction was performed using TaqMan® Probe-Based Gene Expression Assays supplied by Applied Biosystems, Life Technologies (Carlsbad, CA, USA). Individual TaqMan gene expression assays were selected for proinflammatory cytokines (IL-1*β*, IL-6, IL-18, TNF*α*, and HMGB1), for inducible nitric oxide synthase (iNOS) and for NF-*κ*B. Quantitative real-time PCR was performed using 50 ng of cDNA per reaction well on a StepOnePlus™ Real-Time PCR System (Applied Biosystems, Life Technologies, Carlsbad, CA, USA). Gene expression data were normalized to *β*-actin RNA housekeeping gene and calibrated to the control samples (CTL at time 0) according to the ΔΔCT method as previously described [14].

### 2.9. Western Blot Analysis

Immunoblot analysis of murine peritoneal macrophages at 0, 8, and 24 hours post asbestos exposure was performed as previously described [21] using primary antibodies against NLRP3 (catalogue number 15101S, Cell Signaling Technology, Danvers, MA, USA) and iNOS (catalogue number 13120, Cell Signaling Technology, Danvers, MA, USA). Membranes were developed using Western Lighting Chemiluminescence Reagent Plus (PerkinElmer Life Sciences, Boston, MA, USA) and quantified by densitometric analysis of 110 kDa band for NLRP3 and 130 kDa band for iNOS. Densitometric analysis of western blots with *β*-actin normalization of protein expression was performed using Gel-Pro Analyzer software (version 6.0, MediaCybernetics, Silver Spring, MD, USA).

### 2.10. Statistical Analysis

All data were analyzed using two-way analysis of variance (ANOVA) to test for the main effects of time and treatment on study outcome measures. Posttests (Tukey's multiple comparison tests) were conducted analyzing significant differences among treatment groups (CTL, LGM2605, ASB, and ASB + LGM2605) within each respective time point. Statistically significant differences were determined using GraphPad Prism version 6.00 for Windows, GraphPad Software, La Jolla, California, USA. Results are reported as mean ± the standard error of the mean (SEM) from three separate experiments. Levels of target gene mRNA are reported as the mean fold change ± SEM from CTL macrophages at time 0 (not exposed to asbestos and not treated with LGM2605). Statistically significant differences were determined at *p* value < 0.05.

## 3. Results

To determine the usefulness of LGM2605 in preventing asbestos-induced inflammation and oxidative cell damage, we utilized elicited murine peritoneal macrophages (MFs) as a model of tissue phagocyte response to the presence of asbestos in the pleural space using a regimen as outlined in Figure 1().

### 3.1. LGM2605 Blunts the Asbestos-Induced Expression of the Inflammasome

**Figure 2:**
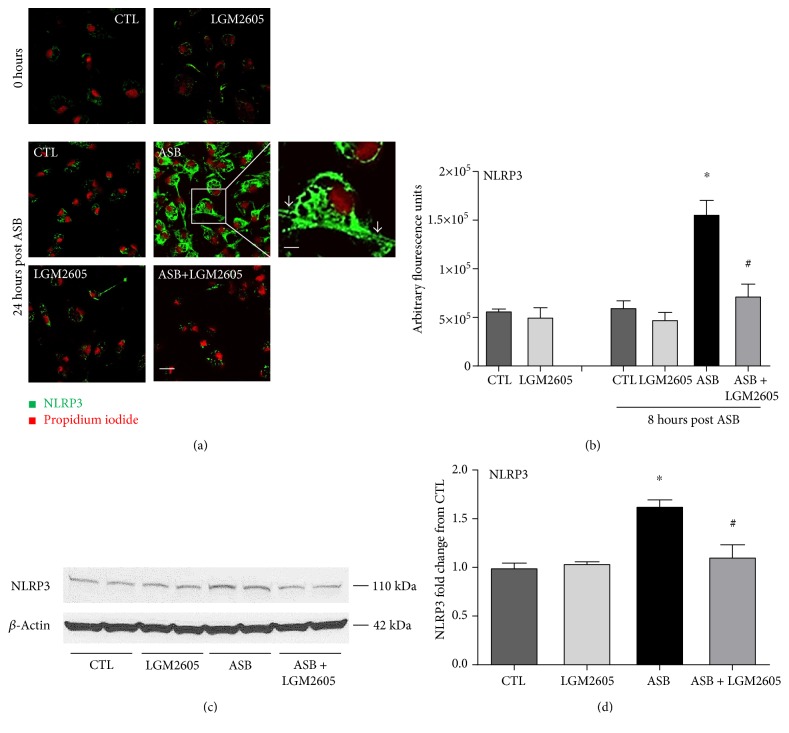
LGM2605 blocks the induction of the NLRP3 subunit of the inflammasome. Murine peritoneal macrophages exposed to LGM2605 4 hours prior to exposure to asbestos fibers were assessed for NLRP3 inflammasome by monitoring the induction of the NLRP3 subunit (green fluorescence) by laser scanning fluorescence microscopy at 24 hours as compared to 0 hours (a). A nuclear stain in the form of propidium iodide (PI) was used to delineate the cells. Magnification 200x; scale is 10 *μ*m. Enlarged inset is at magnification 600x; scale is 30 *μ*m. Grey arrows indicate asbestos fiber engulfed by the macrophage. Quantification of NLRP3 immunostaining (b). Date are presented as arbitrary fluorescence units and as mean ± SEM. Protein levels of the NLRP3 subunit of the inflammasome were evaluated by Western blotting for NLRP3 (c and d). (c) depicts a representative Western blot from three separate experiments at 24 hours. (d) displays mean ± SEM fold change of NLRP3 from CTL. ^∗^Statistically significant difference (*p* < 0.05) between ASB- and CTL-treated cells. ^#^Statistically significant difference (*p* < 0.05) between ASB- and ASB + LGM2605-treated cells.

The NLRP3 subunit of the inflammasome was observed to be expressed at low levels in naïve cells (green fluorescence). Upon asbestos treatment, NLRP3 expression increased significantly (*p* < 0.05) as observed from the increased green fluorescence intensity in cells (Figures 2(a) and 2(b)). Green fluorescence was reflective of the expression of the NLRP3 subunit, while propidium iodide was used to delineate the nuclei of the cells. The fluorescence signal was along the cell membrane and also within intracellular structures. Pretreatment with LGM2605 reduced NLRP3 expression (by 40–81%) to levels comparable to untreated cells.

Induction of the inflammasome by asbestos and inhibition by LGM2605 were also confirmed by Western blotting (Figures 2(c) and 2(d)). Specifically, levels of NLRP3 increased on average 1.63 ± 0.06-fold over control at 24 hours post asbestos exposure. LGM2605 treatment significantly (*p* < 0.01) reduced asbestos-induced NLRP3 expression (1.11 ± 0.09-fold over control). Data are presented as mean ± SEM.

### 3.2. LGM2605 Blunts the Asbestos-Induced Release and Expression of Inflammasome-Activated Cytokines

**Figure 3:**
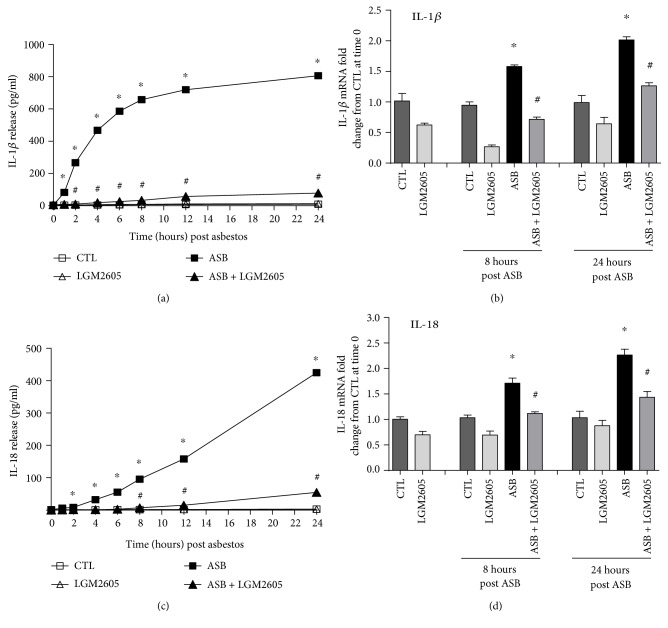
Inflammasome activation following asbestos exposure leads to IL-1*β* and IL-18 secretion via activated caspase-1. Release of IL-1*β* (a) and IL-18 (c) was determined at 0, 1, 2, 4, 6, 8, 12, and 24 hours post asbestos exposure. Samples were run undiluted in triplicate, and cytokine concentrations (pg/ml) are presented as mean ± SEM. Macrophage mRNA expression of IL-1*β* (b) and IL-18 (d) was determined at 0, 8, and 24 hours post asbestos exposure using qPCR. Levels of target gene mRNA were normalized to 18S ribosomal RNA, and values are expressed as mean fold change from CTL at time 0. Data are presented as mean ± SEM. ^∗^Statistically significant difference (*p* < 0.05) between ASB- and CTL-treated cells. ^#^Statistically significant difference (*p* < 0.05) between ASB- and ASB + LGM2605-treated cells.

Asbestos exposure has been reported to activate the NLRP3 inflammasome and lead to the production and release of proinflammatory cytokines, IL-1*β* and IL-18 [11, 12]. Levels of IL-1*β* and IL-18 thus were determined up to 24 hours post asbestos exposure, along with their respective gene expression levels (Figure 3()). Minimal IL-1*β* or IL-18 was released by control (nonasbestos treated) cells or by cells treated with LMG2605 alone. Levels of IL-1*β* rapidly increased within the first 6 hours post asbestos exposure (from 4.04 ± 0.13 pg/ml at baseline to 586.13 ± 4.61 pg/ml) and then plateaued through 24 hours (Figure 3(a)). Levels of IL-18 increased linearly over time up to 24 hours post asbestos (from 1.65 ± 0.10 pg/ml at baseline to 424.62 ± 8.80 pg/ml) (Figure 3(c)). Pretreatment with LGM2605 significantly (*p* < 0.0001) reduced levels of IL-1*β* (Figure 3()) and IL-18 (Figure 3(c)) by 89–96% and 84–95%, respectively. We also determined mRNA levels of IL-1*β* and IL-18 from treated macrophages at 8 and 24 hours post asbestos exposure. Although gene expression levels of both IL-1*β* and Il-18 were elevated (mean fold change ranging from 1.58- to 2.26-fold increase from CTL at time 0), treatment with LGM2605 significantly (*p* < 0.05) reduced levels of both IL-1*β* and Il-18 (by ~89–96%), similar to baseline values (Figures 3(b) and 3(d)). Data are presented as mean ± SEM as well as activated NF-*κ*B by 48-49% while decreasing levels of nitrates/nitrites by 85–93%.

### 3.3. Asbestos-Induced Proinflammatory Cytokine Release and Expression Is Ameliorated by LGM2605

**Figure 4:**
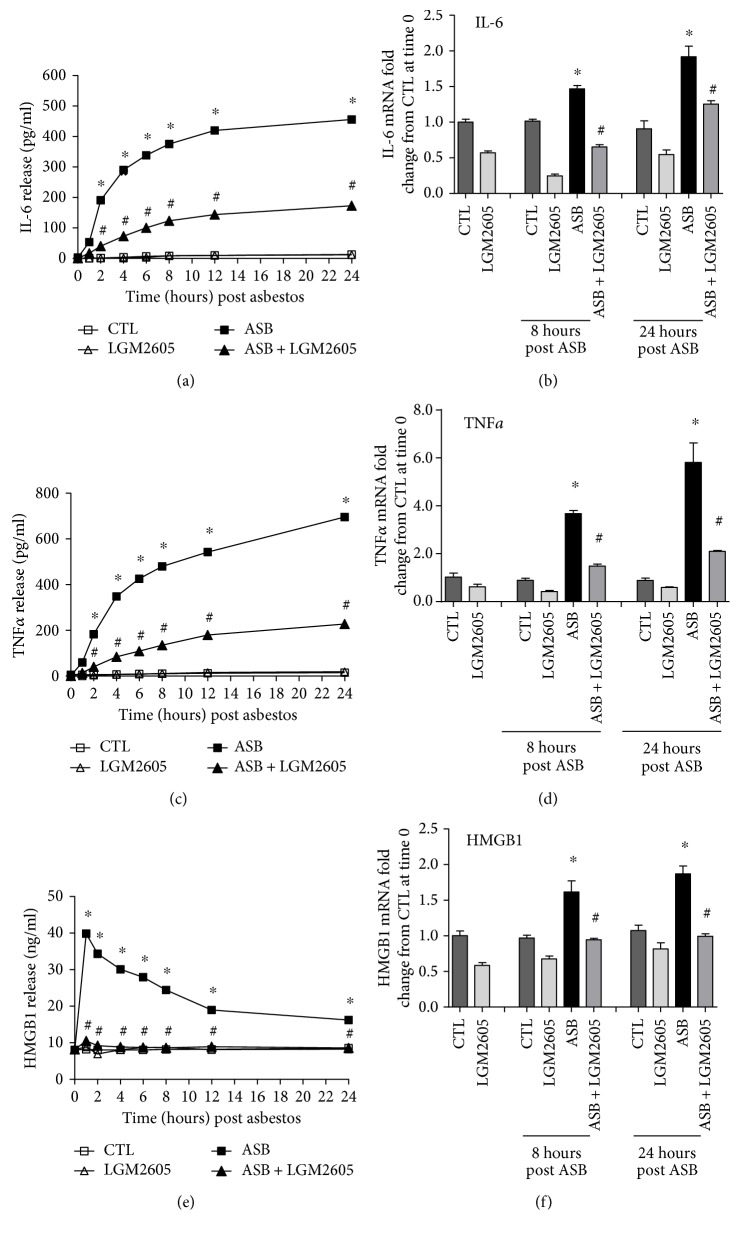
Asbestos-induced proinflammatory cytokine secretion is blunted by LGM2605. Release of IL-6 (a), TNF*α* (c), and HMGB1 (e) was determined at 0, 1, 2, 4, 6, 8, 12, and 24 hours post asbestos exposure. Samples were run undiluted in triplicate, and cytokine concentrations (pg/ml for IL-6 and TNF*α* and ng/ml for HMGB1) are presented as mean ± SEM. Macrophage mRNA expression of IL-6 (b), TNF*α* (d), and HMGB1 (f) was determined at 0, 8, and 24 hours post asbestos exposure using qPCR. Levels of target gene mRNA were normalized to *β*-actin RNA, and values are expressed as mean fold change from CTL at time 0. Data are presented as mean ± SEM. ^∗^Statistically significant difference (*p* < 0.05) between ASB- and CTL-treated cells. ^#^Statistically significant difference (*p* < 0.05) between ASB- and ASB + LGM2605-treated cells.

The inflammatory response post asbestos exposure was further characterized by determining the protein and mRNA levels of the proinflammatory cytokines IL-6, TNF*α*, and HMGB1 (Figure 4()). Minimal amounts of IL-6, TNF*α*, and HMGB1 were released by control (nonasbestos treated) cells or by cells treated with LMG2605 alone. Protein levels of IL-6 (Figure 4(a)) and TNF*α* (Figure 4(c)) peaked at 24 hours post asbestos exposure (455.99 ± 3.03 and 695.34 ± 5.80 pg/ml, resp.) and were significantly (*p* < 0.0001) reduced by 62 and 66%, respectively, among macrophages treated with LGM2605 (172.98 ± 2.76 and 227.89 ± 3.30 pg/ml, resp.). Although the asbestos-induced increase in levels of IL-6 and TNF*α* followed similar kinetics, levels of HMGB1 peaked 30 minutes post asbestos exposure (39.85 ± 1.12 ng/ml) and gradually decreased over time (16.24 ± 0.25 ng/ml at 24 hours post asbestos) (Figure 4(e)). The initial increase in HMGB1 at 30 minutes post asbestos exposure was significantly (*p* < 0.0001) reduced by LGM2605 (ranging from 73 to 75%). Additionally, although levels of IL-6 (Figure 4(b)), TNF*α* (Figure 4(d)), and HMGB1 (Figure 4(f)) mRNA from asbestos-exposed macrophages were significantly (*p* < 0.05) elevated (1.92-, 5.81-, and 1.87-fold) from untreated macrophages at 24 hours post asbestos exposure, mRNA levels from LGM2605-treated macrophages (1.25-, 2.10-, and 1.00-fold from control, resp.) were significantly (*p* < 0.05) decreased from mRNA levels of asbestos-only-exposed macrophages. Data are presented as mean ± SEM.

### 3.4. LGM2605 Prevents Asbestos-Induced Oxidative/Nitrosative Stress and Activation of NF-*κ*B

**Figure 5:**
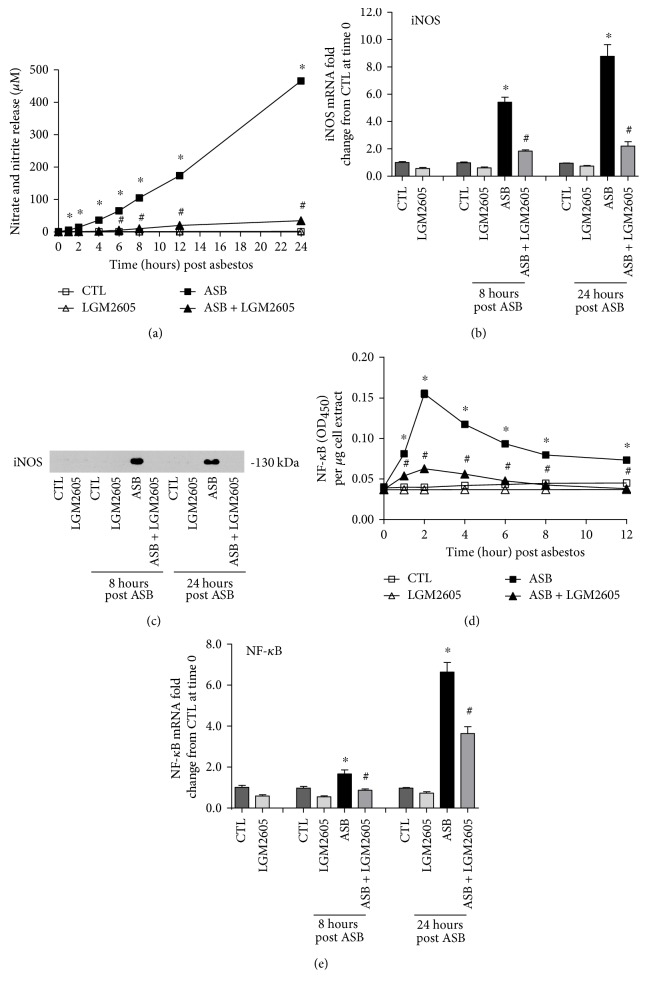
LGM2605 inhibits NF-*κ*B expression and prevents asbestos-induced iNOS expression and nitric oxide production by murine peritoneal macrophages. The concentrations (*μ*M) of nitrates and nitrites (a) were determined in cell culture medium at 0, 1, 2, 4, 6, 8, 12, and 24 hours post asbestos exposure. Macrophage mRNA expression iNOS (b) was determined at 0, 8, and 24 hours post asbestos exposure using qPCR and protein levels of iNOS were evaluated by Western blotting for iNOS (c) (molecular weight 130 kDa). Levels of active nuclear NF-*κ*B (d) were determined at 0, 1, 2, 4, 6, 8, and 12 hours post asbestos exposure, while mRNA expression of NF-*κ*B (e) was determined at 0, 8, and 24 hours post asbestos exposure using qPCR. Levels of target gene mRNA were normalized to *β*-actin RNA, and values are expressed as mean fold change from CTL at time 0. Data are presented as mean ± SEM. ^∗^Statistically significant difference (*p* < 0.05) between ASB- and CTL-treated cells. ^#^Statistically significant difference (*p* < 0.05) between ASB- and ASB + LGM2605-treated cells.

We evaluated levels of total nitrates and nitrites in the cell culture medium as a marker of nitrosative stress following asbestos exposure (Figure 5(a)). Minimal nitrates/nitrites were released by control (nonasbestos treated) cells or by cells treated with LMG2605 alone. Asbestos exposure led to a significant increase in the concentration of nitrates/nitrites (465.99 ± 4.20 *μ*M at 24 hours post asbestos) that was significantly blunted (by 85–93%) by the administration of LGM2605 (34.87 ± 0.55 *μ*M (Figure 5(a)).

We further investigated the observation of decreased nitrosative stress with LGM2605 treatment by determining protein and mRNA levels of inducible nitric oxide synthase in asbestos-exposed macrophages. After 8 and 24 hours of exposure to asbestos, mRNA levels of iNOS were significantly (*p* < 0.05) elevated above untreated macrophages (5.41 ± 0.38- and 8.78 ± 0.85-fold change, resp.) (Figure 5(b)). Alternatively, iNOS gene expression was significantly decreased among LGM2605-treated macrophages exposed to asbestos at both 8 and 24 hours post asbestos (1.84 ± 0.09- and 2.20 ± 0.32-fold change, resp.). Furthermore, although we were able to detect levels of iNOS in macrophages exposed to crocidolite asbestos fibers, iNOS was not detectable in asbestos-exposed macrophages treated with LGM2605 (Figure 5(c)).

The observed decreased expression of iNOS prompted us to further explore molecular targets upstream of iNOS that may be altered by LGM2605. We measured levels of active NF-*κ*B in macrophage nuclear extracts and determined the kinetics of NF-*κ*B nuclear accumulation following asbestos exposure. Following asbestos exposure, we saw significantly (*p* < 0.0001) increased levels of NF-*κ*B present in nuclear extracts from asbestos-exposed macrophages, with the highest concentration occurring after 2 hours postexposure (Figure 5(d)). Gene expression levels of nuclear NF-*κ*B were further induced by asbestos when measured after 8 and 24 hours of exposure (1.67 ± 0.20- and 6.64 ± 0.47-fold change, resp.) (Figure 5(e)). Importantly, treatment of macrophages with LGM2605 significantly (*p* < 0.05) reduced asbestos-induced activation and expression of NF-*κ*B (by ~48%).

## 4. Discussion

Asbestos exposure is a well-established driver of malignant mesothelioma (MM) via an inflammation cascade. Chronic inflammation is believed to play a critical role in the onset and development of MM [6, 9]. In recent years, it has become clear that one of the key inflammatory moieties driving asbestos-induced damage and fibrosis is the induction of the inflammasome [10–13]. As there is no current cure for asbestos-related lung/pleural diseases, blocking the inflammasome may be a potential strategy to reduce the onset of inflammation [23].

Asbestos fibers have been shown to participate in redox reactions to generate several free radicals, including hydroxyl radicals, generated either through a redox reaction or by catalyzing a Fenton-like reaction in exposed cells [24]. These species, collectively called ROS, induce direct oxidative and nitrosative stress besides activating inflammation-signaling pathways. Asbestos fiber internalization generates a significant increase in intracellular ROS, and there is considerable evidence that asbestos-initiated chronic oxidative stress contributes to carcinogenesis and fibrosis by promoting oxidative DNA damage and regulating redox signaling pathways in exposed cells [25].

LGM2605 is a synthetic version of the lignan secoisolariciresinol diglucoside (SDG) which is derived from the natural plant flaxseed. After ingestion, SDG is converted to secoisolariciresinol, which is further metabolized to the entero lignans enterodiol and enterolactone. Our previous work on SDG (and SDG metabolites) shows that this compound can provide protection against oxidative lung injury via multiple mechanisms such as its ability to scavenge ROS and other active radicals and upregulate antioxidant genes via induction of Nrf2 (the transcription factor that regulates antioxidant defense), as well as by reducing the expression and activity of proinflammatory moieties [14, 19, 26].

Our earlier *in vivo* study showed that an SDG-rich formulation given in the diet reduced abdominal inflammation in asbestos-exposed mice. This work is a follow-up investigation to evaluate the mechanisms by which SDG (LGM2605) reduces inflammation *in vivo*. We used murine peritoneal macrophages as an *in vitro* model, as peritoneal macrophages are the major cell type that contributes to both local and systemic inflammatory responses upon contact with foreign agents/pathogens. In this capacity, these cells often drive various inflammatory pathologies. Furthermore, they are involved in the clearance of foreign particles and cellular debris, as well as pathogenic agents. The dose of LGM2605 used here was 50 *μ*M based on an earlier study where this dose was sufficient to scavenge ROS in asbestos-treated macrophages [14]. Even lower doses (<1 *μ*M) were shown to be effective in ROS scavenging, such as ROS generated from radiation exposure of cells [17].

**Figure 6:**
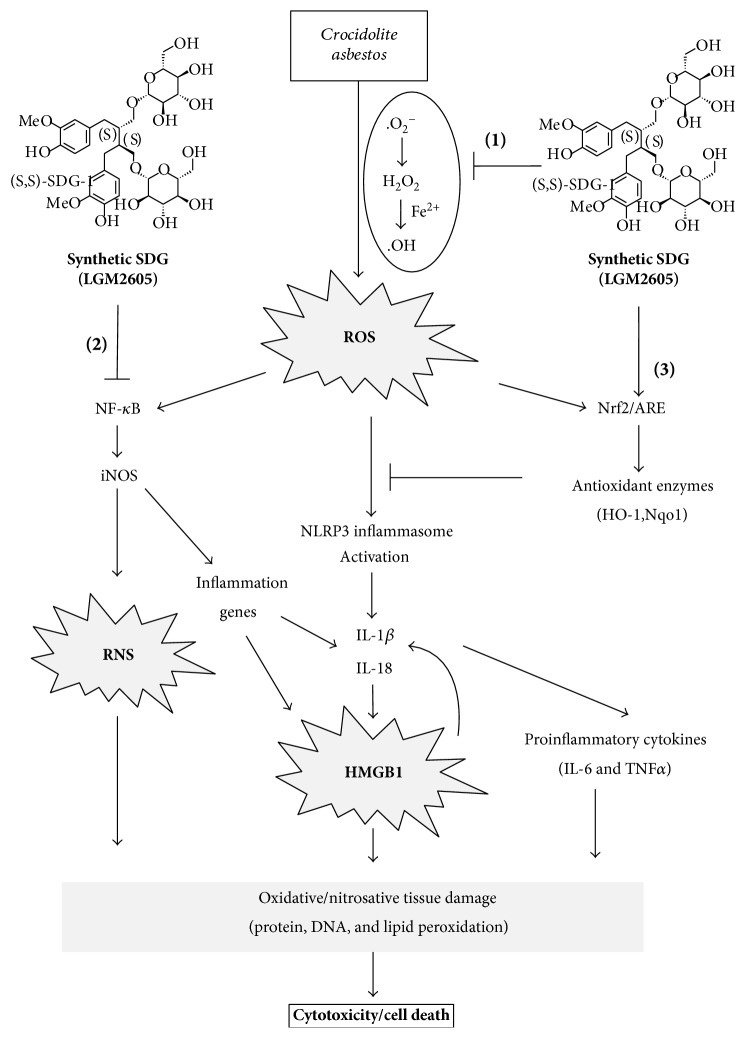
Role of the LGM2605 in preventing asbestos-induced inflammation, oxidative/nitrosative cell damage, cell injury, and cell death proposed mechanism of asbestos-induced inflammation and oxidative/nitrosative stress and the protective effect of LGM2605. Asbestos exposure leads to the production of ROS (such as H_2_O_2_, ^.^OH) that activates an inflammatory cascade, of which the NLRP3 inflammasome is involved. NLRP3 activation leads to the release of cytokines IL-1*β* and IL-18. These cytokines drive inflammation-induced cell death. Under these conditions, the damage-associated molecular pattern protein HMGB1 is either released by dead cells or passively secreted from inflamed cells. HMGB1 release can also lead to a feed forward cycle of IL-1*β* and IL-18 induction and activation. Importantly, asbestos-induced inflammation is largely driven by HMGB1 and the NLRP3 inflammasome. Frustrated phagocytosis of asbestos fibers may lead to cell necrosis and the subsequent release of HMGB1, which promotes the activation of the NLRP3 inflammasome. Asbestos-induced ROS generation exacerbates this signaling cascade and further promotes malignant transformation. Combined, HMGB1 and NLRP3 inflammasome activation, induces a proinflammatory signaling cascade that ultimately leads to IL-1*β* and TNF*α* secretion and NF-*κ*B activation. LGM2605 exhibits a pluripotent role in preventing ROS/RNS generation and inflammation following asbestos exposure through several mechanisms: (1) LGM2605 directly scavenges free radicals (ROS, such as H_2_O_2_, ^.^OH) and mitigates asbestos-induced ROS/RNS generation, (2) LGM2605 inhibits the proinflammatory NF-*κ*B pathway presumably via reduced levels of ROS (either due to direct scavenging or due to increased antioxidant enzyme expression) or via direct inhibition of the transcription factor. LGM2605 decreased the levels of NLRP3 inflammasome (protein), HMGB1 (protein and mRNA), and the levels of iNOS (protein and mRNA), and (3) LGM2605 activates Nrf2 and induces the expression of cellular antioxidant and detoxification enzymes.

Our findings highlight the pluripotent properties of LGM2605 (see Figure 6) in an *in vitro* model of asbestos exposure. Specifically, redox signaling following asbestos exposure is thought to occur through 3 primary mechanisms: (1) ROS release from macrophages following frustrated phagocytosis of the asbestos fiber [27], (2) generation of reactive oxygen species (especially the DNA-damaging hydroxyl radical) due to the high iron content (~27–30%) of crocidolite asbestos [28, 29] that can be redox activated, and (3) mitochondrial-derived ROS release from macrophages following asbestos exposure. Furthermore, asbestos fibers have been shown to induce the generation of hydrogen peroxide, superoxide radical, and reactive nitrogen species that may lead to both extracellular cell signaling (ROS interaction with the TNF receptor) and intracellular cell signaling (NLRP3 inflammasome activation).

Thus, in the current study, we observed the ability of LGM2605 to blunt asbestos-induced inflammation and alter the inflammatory processes that contribute to cytokine production and secretion. Importantly, LGM2605 inhibited activation of NF-*κ*B although the mechanism by which this occurs is not clear. We presume that reduced activation of NF-*κ*B is via the scavenging of ROS (either directly or by increased cellular antioxidant status) or via inhibiting the transcription factor itself. Inhibition of NF-*κ*B caused the lowering of key inflammasome-activated cytokines IL-1*β* and IL-18, suggesting further modulation of the NLRP3 inflammasome by LGM2605. LGM2605 reduced HMGB1 (protein and mRNA) levels indicating that HMGB1 induced the secretion of inflammatory cytokines. Asbestos-induced ROS generation may lead to NF-*κ*B activation through ROS signaling via the TNF receptor. Asbestos induces a multitude of redox cell signaling pathways through direct interaction of the asbestos fiber with the cell membrane, extracellular ROS generation and interaction with cell receptors, and intracellular ROS generation. Ultimately, the cell signaling pathways influenced by asbestos fiber exposure are key pathways implicated in gene expression and cell fate. The inflammatory cascade initiated within the macrophage involves both asbestos-induced NALP3 inflammasome activation and ROS-induced TNF*α* and NF-*κ*B signaling. Secretion of IL-1*β* and TNF*α* is directly implicated in asbestos-induced carcinogenesis. Pretreatment with LGM2605 not only decreases asbestos-induced ROS generation but also reduces levels of HMGB1 and TNF*α*, which are implicated in NF-*κ*B activation.

Additionally, LGM2605 reduced levels of asbestos-induced oxidative and nitrosative stress by decreasing expression of iNOS and enhancing levels of key antioxidant enzymes involved in the detoxification of reactive oxygen species. We have previously reported on the ROS scavenging ability of LGM2605 in asbestos-exposed macrophages [14]. Specifically, LGM2605 significantly reduced asbestos-induced ROS and upregulated expression of Nrf2 phase II detoxification enzymes, ultimately reducing cellular injury and cell death indicated by LDH release.

Based on our earlier work, it is clear that several free radicals (H_2_O_2_, O_2_, and ·OH), collectively called ROS, are scavenged by LGM2605 in cell-free systems. In asbestos-treated cells, we have previously reported a decrease in H_2_O_2_ production [14], but the effect on other radicals per se was not studied. The contribution of peroxynitrite versus ROS-induced damage with asbestos treatment is also not very clear. However, based on our studies, it emerges that LGM2605 is protective against asbestos-induced damage by blocking key elements of oxidative and nitrosative stress that have been reported elsewhere to participate in inflammation, cell death, and tissue damage.

Asbestos exposure leads to increases in the levels of ROS, such as superoxide anion (O_2_−·), hydroxyl radical (·OH), and hydrogen peroxide (H_2_O_2_) both inside the cell and in the extracellular matrix due to the surface reactivity of the crocidolite asbestos. The hydroxyl radical (·OH) scavenging ability of SDG has previously been reported by Prasad [30, 31]. Furthermore, Kitts and colleagues evaluated the hydroxyl and peroxyl radical scavenging activity of SDG [32] and reported on the effectiveness of SDG in reducing lipid peroxidation and deoxyribose oxidation. Hu et al. reported on the effectiveness of SDG against 1,1-diphenyl-2-picrylhydrazyl (DPPH·) and 2,2′-azo-bis (2-amidinopropane) dihydrochloride (AAPH) peroxyl radicals [33]. Furthermore, we have previously reported on the antioxidant properties of synthetic SDG (LGM2605) showing powerful scavenging activity against hydroxyl radicals, peroxyl radicals, and DPPH radicals [18].

Taken together, via a multiprong mechanism of action described in Figure 6, LGM2605 reduced asbestos-induced NLRP3 inflammasome expression, proinflammatory cytokine release, and markers of injury and nitrosative stress in murine peritoneal macrophages supporting its possible use in preventing the asbestos-induced inflammatory cascade. Combined, our findings support the usefulness of LGM2605 as a potential chemopreventive agent in reducing the early inflammatory and cytotoxic effects that occur following asbestos exposure.

SDG, an antioxidant isolated from flaxseed, is metabolized by intestinal bacteria to enterodiol (ED) and enterolactone (EL) which are bioactive. However, SDG also has strong direct antioxidant properties *in vitro* without the need for metabolic activation [17]. The antioxidant activities of these three lignans (SDG, EL, and ED) were shown by their ability to inhibit linoleic acid lipid peroxidation, indicating direct hydroxyl radical scavenging activity [31, 32]. Since oxidant stress is implicated in the etiology of cancer, the therapeutic or preventive use of dietary flaxseed or flaxseed-derived lignans has been considered in certain malignancies such as in lung cancer [34, 35].

Our initial findings showed that treatment with the LGM2605 significantly inhibited the release of inflammasome-activated cytokines, IL-1*β* and IL-18. However, macrophages treated with LGM2605 also displayed significantly reduced levels of other proinflammatory cytokines, such as TNF*α* and IL-6, suggesting that both the NF-*κ*B system and NLRP3 inflammasome pathway are blocked by LGM2605. Additionally, LGM2605 blocked the asbestos-induced release of HMGB1, which is either released passively by dead cells or secreted actively by stressed cells. Our earlier data showed that asbestos induces cell death (LDH release) which is ameliorated by the action of LGM2605, so clearly, cell death does presumably play a role in the increase of HMGB1 levels. Our current study shows HMGB1 mRNA and protein levels declined with LGM2605 treatment indicating that this agent blocks the induction of this danger-associated molecular pattern (DAMP) protein (Figures 4(e) and 4(f)). However, regardless of the mechanism of its release, HMGB1 outside the cell behaves as a DAMP protein or alarmin that activates the innate immune system either alone or in conjunction with cytokines and appears to be critical in the malignant transformation of mesothelial cells. HMGB1 amplifies the inflammatory response in general (by chemotaxing leukocytes (including neutrophils and mast cells [36]) and activating NF-*κ*B) but also serves as an important protumorigenic cytokine that enhances the growth, survival, and invasiveness of the mesothelial cells [1]. Therefore, the fact that LGM2605 reduces HMGB1 via either reducing its release by stressed cells or by reducing cell death implies that this agent (LGM2605) can effectively “block” the asbestos-induced inflammatory signaling cascade that leads to activation of innate immune response and subsequent malignant transformation.

The activation of the NLRP3 inflammasome that drives maturation of proinflammatory cytokines such as interleukin-1*β* (IL-1*β*) and IL-18, and leads to cell death (pyroptosis) has been reported elsewhere to be ROS regulated. Indeed, ROS have been reported to be crucial in triggering NLRP3 inflammasome formation and activation in response to both exogenous stimuli and endogenous signals in the form of damage-associated molecular patterns secreted by apoptotic cells [37]. Inhibition of NADPH oxidase-derived ROS prevented ATP-induced caspase-1 activation and IL-1*β* production in alveolar macrophages [38].

Our previous work shows increased ROS generation following asbestos exposure, which presumably participates in NLRP3 induction, as pretreatment with the ROS scavenger LGM2605, which reduced its activation as monitored by IL-1*β* and IL-18. Earlier studies reported the importance of NADPH oxidase-derived ROS in activating NLRP3 in response to ATP, asbestos, and silica [11]. Indeed, monocyte THP-1 cells that produced caspase 1 (i.e., showed NLRP3 inflammasome activation) in response to asbestos or silica showed a blunted response in specific knockdown of NADPH oxidase subunit p22phox or when treated with ROS scavengers (N-acetylcysteine and ammonium pyrrolidine dithiocarbamate) [11].

While induction and activation of the NLRP3 inflammasome are accepted to be redox regulated, the exact mechanism by which this occurs is not clear. One possibility is via the thioredoxin-interacting protein (TXNIP) that has been shown elsewhere to be associated with NLRP3 as a binding partner in a NLRP3-TXNIP complex [39]. TXNIP is a negative regulator of the antioxidant thioredoxin (TRX), and the dissociation of TXNIP from TRX is a ROS dependent process [39]. Thus, high levels of ROS lead to dissociation of TXNIP from TRX thus potentially allowing TXNIP to bind to NLRP3.

We report the ability of LGM2605 to both scavenge free radicals and detoxify ROS through direct and indirect molecular effects. We have previously reported the direct free radical scavenging ability of LGM2605 in a murine endothelial cell model of gamma radiation-induced ROS [17] and, therefore, studied this potential mechanism in our system. As shown in Figure 6, LGM2605 likely acted as a direct free radical scavenger and antioxidant in a dose-responsive manner. In addition to the direct free radical scavenging ability of LGM2605, we have shown that flaxseed, and its bioactive lignan component, can activate Nrf2 [16, 17, 21, 40]. Nrf2 is a master transcriptional regulator of carcinogen detoxifying and antioxidant enzymes (such as HO1 and NQO1) and plays a major role in tissue protection. These findings are in agreement with those of Velalopoulou et al., where LGM2605 protected normal lung cells from radiation-induced DNA damage through direct free radical scavenging and boosting of endogenous antioxidant enzyme gene expression [21].

## 5. Conclusion

An ideal agent used for the chemoprevention of asbestos-induced mesothelioma must be nontoxic, tolerable, and effective in interfering in asbestos-induced carcinogenesis. LGM2605 reduced proinflammatory cytokine release and markers of oxidative and nitrosative stress in murine peritoneal macrophages and may impede the asbestos-induced inflammatory cascade on the way to malignancy. Importantly, the ability of LGM2605 to interfere in multiple molecular pathways (boosting antioxidant defenses and blocking inflammation) provides strong evidence for its potential usefulness in chronic *in vivo* models of asbestos-induced mesothelioma.

## Abbreviations

ASB: Asbestos
CTL: Control
DAMP: Danger-associated molecular pattern
ED: Enterodiol
EL: Enterolactone
ELISA: Enzyme-linked immunosorbent assays
FLC: Flaxseed lignan component
HMGB1: High mobility group box 1
HO-1: Heme oxygenase-1
IACUC: Institutional Animal Care and Use Committee
IL-1*β:*: Interleukin-1*β*
IL-6: Interleukin-6
IL-18: Interleukin-18
iNOS: Inducible nitric oxide synthase
IP: Intraperitoneal
LC/MS/MS: Liquid chromatography/tandem mass spectrometry
LDH: Lactate dehydrogenase
LGM2605: Synthetic secoisolariciresinol diglucoside
MF: Macrophages
MDA: Malondialdehyde
MM: Malignant mesothelioma
NF-*κ*B: Nuclear factor kappa-light-chain-enhancer of activated B cells
Nrf2: Nuclear factor E2-related factor 2
Nqo1: NADPH:quinone oxidoreductase-1
PBS: Phosphate-buffered saline
PL: Peritoneal lavage
PLF: Peritoneal lavage fluid
qPCR: Quantitative polymerase chain reaction
RNS: Reactive nitrogen species
ROS: Reactive oxygen species
SDG: Secoisolariciresinol diglucoside
TNF*α:*: Tumor necrosis factor alpha
WBC: White blood cells.
